# *Pisinnocaris subconigera*—a valid species of early Cambrian fuxianhuiid

**DOI:** 10.7717/peerj.20483

**Published:** 2026-02-03

**Authors:** Huijuan Mai, Hong Chen, Ailin Chen, Jin Guo, Xianguang Hou, Yu Liu

**Affiliations:** 1 Yunnan Key Laboratory for Palaeobiology, Institute of Palaeontology, Yunnan University, Kunming, Yunnan, China; 2 MEC International Joint Laboratory for Palaeobiology and Palaeoenvironment, Yunnan University, Kunming, Yunnan, China; 3 School of Biological Sciences and Technology, Liupanshui Normal University, Liupanshui, Guizhou, China; 4 Guizhou Provincial Key Laboratory for Palaeontology and Palaeoenvironment, Guizhou University, Guiyang, Guizhou, China; 5 Research Center of Paleobiology, Yuxi Normal University, Yuxi, Yunnan, China; 6 Chengjiang Fossil Museum of the Management Committee of the Chengjiang World Heritage Fossil Site, Chengjiang, Yunnan, China; 7 Southwest United Graduate School, Kunming, Yunnan, China; 8 School of Geography, Geology and the Environment, University of Leicester, Leicester, United Kingdom

**Keywords:** Cambrian, Chengjiang biota, Fuxianhuiids, Pisinnocaris subconigera

## Abstract

*Pisinnocaris subconigera* was first described as a rare, small euarthropod from the early Cambrian Chengjiang biota, southwestern China. The taxonomic validity of this species was later challenged due to the lack of essential morphological details to differentiate it from *Fuxianhuia protensa*, especially from the juvenile perspective. Here, we examined the holotype and additional specimens of *P. subconigera* with multiple imaging techniques, such as microscope optical imaging, micro-CT scanning and computer-based 3D rendering, and revealed the previously unknown ventral organization of *P. subconigera*. New findings include four short prothoracic segments each bearing a pair of biramous appendages, four opisthothoracic segments each with four pairs of appendages, and four limbless abdominal segments. Both the small and large individuals exhibit unique and consistent morphological characteristics, indicating that *P. subconigera* does not represent the larval or juvenile form of any fuxianhuiid as previously proposed. Combined with phylogenetic analyses, our study suggests that *P. subconigera* remians as a valid member of the early Cambrian fuxianhuiids.

## Introduction

Fuxianhuiids are a group of early Cambrian euarthropods with a unique combination of characters: an eye-bearing anterior sclerite; a pair of antennae; a carapace overlapping a pair of specialized post-antennal appendages (SPAs) and several shortened prothoracic segments each carrying a pair of biramous limbs; opisthothoracic segments bearing more than one pair of biramous limbs and the abdominal segments bearing no limbs (Ortega-Hernández, Yang & Zhang, 2018; Chen et al., 2018). Thus far, all known fuxianhuiids are reported exclusively from the lower Cambrian (Series 2, Stages 3 and 4) in eastern Yunnan, China (Hou, 1987; Hou & Bergström, 1991; Chen, 2005; Luo et al., 2007; Yang et al., 2013, 2018; Chen et al., 2018; Liu et al., 2020a). Despite their limited distribution both in space and time, fuxianhuiids play a role in understanding early arthropod evolution—they have been considered stem-group arthropods based on the relatively simple limb morphology (*e.g*., Waloszek et al., 2005), or the sister-group to crown Mandibulata based on its head segmentation pattern and the morphology of the SPAs (Aria, Zhao & Zhu, 2021). Taxonomic controversies among some fuxianhuiids have also occurred. These include (1) *Shankouia zhenghei* (Waloszek et al., 2005) hypothesized to represent a synonym of *Liangwangshania biloba* (Chen et al., 2018); (2) *Xiaocaris* has been separated from *Jianshania* to represent the latest known genus of fuxianhuiid (Liu et al., 2020a); and (3) *Pisinnocaris subconigera* has been considered a junior synonym of *Fuxianhuia protensa* (Fu et al., 2018).

*Pisinnocaris subconigera*, a rare, small euarthropod from the early Cambrian Chengjiang biota, was first reported by Hou & Bergström (1998), but with uncertainties pertaining to the higher taxonomic assignment (*i.e*., Class, Order and Family) (see Hou & Bergström, 1998). Due to the lack of specimens, *P. subconigera* has only been briefly mentioned in a few studies after its original description (see Xu, 2004; Zeng et al., 2014). In 2018, Fu et al. (2018) identified new individuals of *P. subconigera* preserved with specimens representing various ontogenetic stages of *Fuxianhuia protensa* (Fu et al., 2018). However, we hypothesize that those new individuals of *P. subconigera* are misinterpreted as juveniles of *F. protensa*, which then leads to questions about the taxonomic validity of *P. subconigera* (Fu et al., 2018).

**Figure 1:**
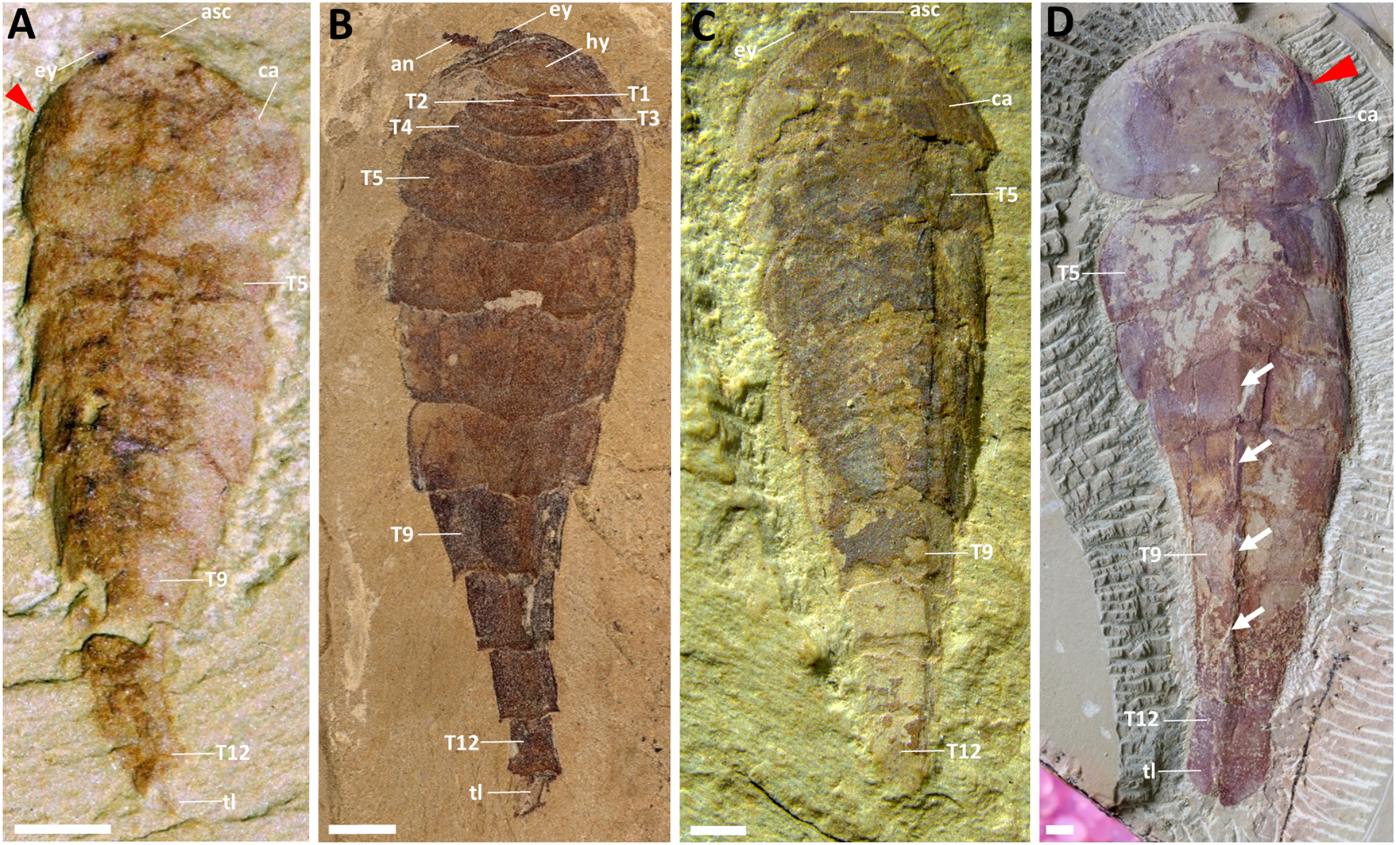
Dorsal morphology of *Pisinnocaris subconigera*. (A) Holotype, NIGPAS 115417a, with four opisthothoracic tergites (T5–T8) and one of the paired leaf-like tail flukes. (B) CJHDM00070, lack of carapace reveals four reduced/shortened prothoracic (T1–T4) tergites underneath. The rest of the body consists of four opisthothoracic (T5–T8) and fourabdominal tergites (T9–T12), and a pair of leaf-shaped tail flukes. (C) YKLP 17301, displays four opisthothoracic tergites (T5–T8). (D) CJHMD00066a, the largest specimen found so far, showing four opisthothoracic tergites (T5–T8) and a pair of leaf-like tail flukes. Arrows indicate keels. Red triangle indicates folds on edges of the carapace. Abbreviations: an, antenna; asc, anterior sclerite; ca, carapace; ey, eye; hy, hypostome; T*n*, trunk segment *n*; tl, tail flukes. Scale bars: 1 mm.

Here, we restudy the holotype specimen of *P. subconigera* together with several new ones collected in recent years (Figs. 1 and S1). Multiple imaging techniques, such as microscope optical imaging, micro-CT scanning and computer-based 3D rendering help reveal new morphologies from the ventral surface of the animal, which provides new evidence to clarify the taxonomic validity and the phylogenetic position of this species.

## Materials and Methods

### Materials

Specimens used in this study include fourteen nearly complete fossils of *P. subconigera*, two slabs with *P. subconigera and F. protensa* preserved together, one fossil of each *Chengjiangocaris longiformis*, *Liangwangshania biloba* and *Guangweicaris spinatus* and three small fossils of unidentified species. These specimens were collected from Chengjiang, Yunnan Province, China, and are deposited either at Yunnan Key Laboratory for Palaeobiology, Yuxi Normal University, or Chengjiang Fossil Museum. Details of the specimens can be found in Table S1. All studied fossils are preserved in yellowish mudstones from the Yu’anshan Member of the Chiungchussu Formation, Cambrian (Series 2, Stage 3), the main localities are shown in Fig. S2 (see also Hou et al., 2017). The specimens are partially pyritized which allows enough signal revealed with micro-computed tomography (micro-CT) scanning (Gabbott et al., 2004).

## Methods

Optical photographs of specimens shown in all figures were captured using a Canon EOS 5DSR with an MP-E 100 mm objective lens.

### Micro-computed tomography (Micro-CT)

X-ray tomography was used to scan three specimens with a Zeiss Xradia Versa 520 micro-CT scanner at the Yunnan Key Laboratory for Palaeobiology, Yunnan University, China. Scanning parameters are as the follows: NIGPAS 115417a (Holotype): beam strength: 50 kV/4w, no filter, pixel size: 3.65 µm, number of TIFF images: 2,799; YKLP 17301: beam strength: 50kV/4w, no filter, pixel size: 6.31 µm, number of TIFF images: 2,435; CJHMD00066a: beam strength: 80 kV/7w, no filter, pixel size: 16.54 µm, number of TIFF images: 3,511. Volumetric reconstructions of specimens from tomographic slices were performed in Drishti software (v 2.4) as described before (Zhang et al., 2022).

### Phylogenetic analyses

Our phylogenetic analyses are based on a previously published character matrix for annelids and their close relatives (Yang et al., 2018), which has been updated with the addition of new taxa and fossil data (Chen, 2020). Analyses are performed with TNT (Maximum Parsimony) and MrBayes (Bayesian Inference) (Ronquist et al., 2012; Du et al., 2019). Parsimony analysis was undertaken with the software TNT (version 1.5), where the traditional search, implied weights and equal weights were used. Other settings included a random seed of 1, k = 3 and a strict consensus rule. Bayesian analysis was conducted in MrBayes (version 3.2.7), use the same matrix but removed six consecutive measurement data (characters 0 to 5, which is non-classified data), with a run of 20.000.000 Markov Chain Monte Carlo generations containing four Markov chains under the MKV+ gamma model. In each run, trees were collected by a sampling frequency of every 1,000 generations and with the first 25% of the samples discarded as burn-in.

## Results

### Systematic Paleontology

Euarthropoda Lankester, 1904

Fuxianhuiida Bousfield, 1995

**Constituent taxa**. From the Chengjiang Biota (Cambrian Series 2, Stage 3): *Fuxianhuia protensa* Hou, 1987, *Chengjiangocaris longiformis* Hou & Bergström, 1991, *Pisinnocaris subconigera* Hou & Bergström, 1998, *Liangwangshania biloba* Chen, 2005, and *Xiaocaris luoi* Liu et al., 2020a; Xiaoshiba Biota (Cambrian Series 2, Stage 3): *Fuxianhuia xiaoshibaensis* Yang et al., 2013, *Chengjiangocaris kunmingensis* Yang et al., 2013, and *Alacaris mirabilis* Yang et al., 2018; Guanshan Biota (Cambrian Series 2, Stage 4): *Guangweicaris spinatus* Luo et al., 2007.

Family incert. Sedis

Genus *Pisinnocaris* Hou & Bergström, 1998

**Type species**. *Pisinnocaris subconigera* Hou & Bergström, 1998; by monotypy.

**Emended diagnosis**. As for type species by monotypy, see below.

**Remarks**. *Pisinnocaris* differs from other fuxianhuiids in the number of shortened prothoracic segments, with four such segments in *Pisinnocaris*, three in *Fuxianhuia* and *Guangweicaris* (Fuxianhuiidae), five in *Chengjiangocaris* and *Alacaris* (Chengjiangocariidae), and six in *Liangwangshania*. Among fuxianhuiids, *Pisinnocaris* has the lowest trunk segment number, only twelve (from prothorax to the posterior end of the body). All the other fuxianhuiids possess over twelve trunk segments, of which *Liangwangshania* bears more than forty trunk segments. *Pisinnocaris* is also unique in its conical body shape.

*Pisinnocaris subconigera* Hou & Bergström, 1998

**Figure 2:**
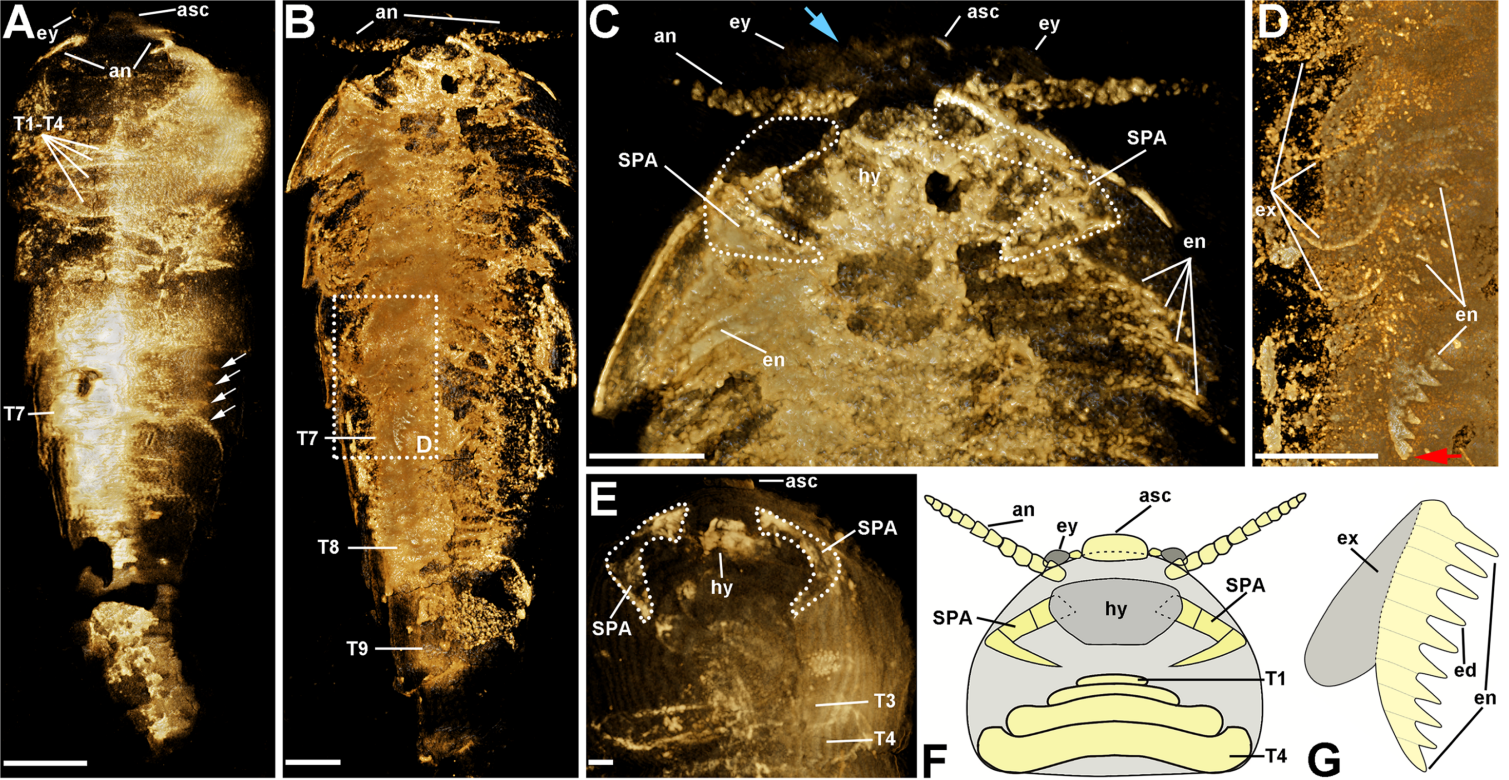
Morphological details of *Pisinnocaris subconigera*. (A) Screenshot of a 3D model derived from a CT scan of the holotype shown in Fig. 1A. Dorsal view showing four anterior shortened segments (T1–T4) and four pairs of appendages (white arrows) associated with T7. (B) Screenshot of a 3D model derived from a CT scan of YKLP 17301 shown in Fig. 1C. Ventral view showing the arrangement of the appendages. Note that there’s no appendage associated with T9 and the subsequent segments. (C) Close-up of the head in B, showing the eyes, the antennae, the SPAs, the hypostome, and the first four pairs of biramous appendages (en). Blue arrowhead indicates the eyestalk. (D) Close-up of the trunk in B, showing four flap-like exopods (ex) preserved on T6, along with an almost complete endopod in T7 (en). Red arrowhead indicates the distal podomere of the endopod in subtriangular shape. (E) Screenshot of a 3D model derived from a CT scan of CJHMD00066a shown in Fig. 1D. Ventral view showing a pair of SPAs with the hypostome (hy) in-between. The last two shortened prothoracic segments (T3, T4) are also detected. (F) Reconstruction of the ventral side of the head. (G) Reconstruction of a biramous appendage consisting of a flap-like exopo d (gray) and an endopod bearing at least ten podomeres (yellow). Abbreviations: an, antenna; asc, anterior sclerite; ca, carapace; ed, endite; en, endopod; ex, exopod; ey, eye; hy, hypostome; SPAs, specialized post-antennal appendages; T*n*, trunk segment *n*. Scale bars: 1 mm.

1998 *Pisinnocaris subconigera* sp. nov.; Hou & Bergström, 1998: 395–401, text-fig. 2.

1999 *Pisinnocaris subconigera* Hou & Bergström, 1998; Hou et al., 1999: 91, pl. 124.

**Figure 3:**
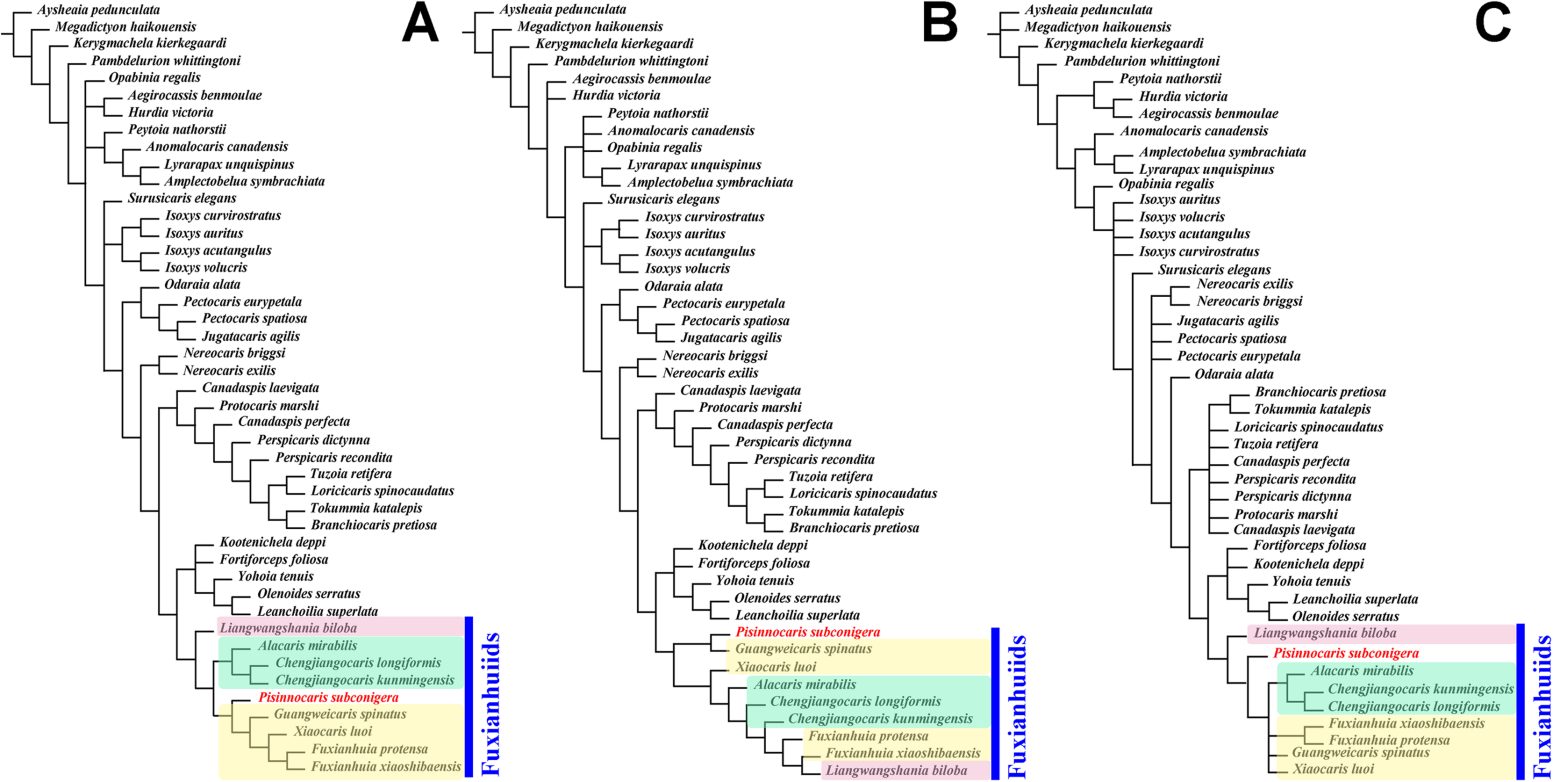
Phylogenetic analysis of fuxianhuiids. (A) Strict consensus of parsimonious trees calculated under implied weights (k = 3). (B) Strict consensus of parsimonious trees calculated under equal weights. (C) Bayesian phylogenetic analyses. A blue bar represents the clade of fuxianhuiids, *P. subconigera* is highlighted in red, the red box highlights that the species has six prothoracic segments, the green box highlights that the species has five prothoracic segments and the yellow box highlights that the species has three prothoracic segments.

2018 *Fuxianhuia protensa* Hou, 1987; Fu et al., 2018, text-figs.1, 3, 13.

**Type material:** Holotype: NIGPAS 115417 (Fig. 1A). A small arthropod with large, subcircular carapace and tapering body with at least ten long tergites *(Hou & Bergström, 1998*).

**Type locality and horizon**: *Eoredlichia* Trilobite Zone of the Yu’anshan Member

(Chiungchussu Formation), Cambrian Series 2, Stage 3, Maotianshan Mountain, eastern Yunnan province.

**Additional material**: Since the original description of this species, few additional specimens have been reported (Xu, 2004; Zeng et al., 2014; Fu et al., 2018). To this we add fourteen specimens from the Xiaolantian section (shown in Fig. S2), China, Cambrian Series 2, Stage 3.

**Emended diagnosis**: Subcircular carapace, posterior margin without depression; anterior sclerite supporting stalked eyes; a pair of specialized post-antennal appendage; four shortened prothoracic segments; four opisthothoracic segments; four limbless abdominal segments; body gradually narrowed, terminated with a pair of leaf-like tail flukes.

## Description

### Dorsal morphology

Our specimens indicate that the body size of *Pisinnocaris subconigera* ranges from 8.92 to 47.62 mm, while the number of trunk segments remains unchanged (Figs. 1 and S1). An anterior sclerite bearing a pair of lateral eyes is located in front of the subcircular carapace that accounts for approximately 1/4–1/3 of the total body length. The anterior margin of the carapace is rounded, with its lateral margins curving inward to form the posterior margin. Unlike in other fuxianhuiids, the median portion of the posterior margin is straight without bending forward. The ratio of the carapace’s width to length is 1.45–1.88, representing the second highest among fuxianhuiids after *F. protensa*. Lateral wrinkles can be formed due to dorsal-ventral compression, indicating a slightly swollen morphology of the front-middle region of the carapace (red triangle indicated in Figs. 1A and 1D). Notably, the carapace is overlapping four shortened prothoracic segments that can only be observed when most of the carapace is missing (Fig. 1B).

The trunk of *Pisinnocaris subconigera* is composed of twelve segments that can be further divided into three tagmata: a prothorax, an opisthothorax, and a limbless abdomen (Fig. 1). The prothorax consists of four shortened segments (T1–T4) whose tergites gradually increase in width and length towards the posterior. The first prothoracic segment is the smallest and can be observed in two specimens: one with the structure fully exposed (Fig. 1B), and the other imaged with CT scanning and 3D rendering techniques (Fig. 2A). Length of this somite is less than 0.1 mm in both specimens. The last two segments of the prothorax (T3, T4) are 5–8 times wider than length and can also be observed in the CT scans of the largest specimen (Fig. 2E). The posterior margin of each preceding segments usually abuts against the anterior part of the following ones.

The opisthothorax comprised of four segments (T5–T8) whose tergites are wider than those of the prothoracic ones, but becomes narrower towards the posterior of the body. The first opisthothoracic segment (*i.e*., T5) bears the widest tergite throughout the entire body (Fig. 1), with a width-to-length ratio of approximately 3.5. Its length is equivalent to all the following tergites. As in the prothorax, the posterior margin of each tergite overlaps the anterior margin of the following one. The overlapping area is approximately 1/3 of the total length of the tergite. Each opisthothoracic tergite bears a keel extending towards the posterior (Fig. 1D). Subsequent segments form the limbless abdomen which tapers and terminates with the last segment forming a sub-square in shape. A pair of leaf-like tail flukes are attached to the last abdominal segment (Figs. 1A and D).

### Ventral morphology

*Pisinnocaris subconigera* bears a pair of small, ovoid eyes each connected to the ventral side of the anterior sclerite *via* a stalk (Figs. 1A–1C; 2A and 2C). Unlike *Fuxianhuia protensa* whose eyes, eye stalks, and anterior sclerite are located in front of the anterior margin of the carapace, these anteriormost structures of *P. subconigera* are partially covered by the carapace and thus less obvious from the dorsal perspective (Figs. 1 and 2). Similar to other fuxianhuiids, *P. subconigera* possesses a pair of antennae each consisting of a minimum of ten antennal podomeres. The proximal podomere is wider than its length, while the subsequent podomeres gradually taper and elongate, so the proximal portion exhibits a moniliform appearance, whereas the distal part displays a claviform shape. No antennal setae have been observed in any specimens studied herein, most likely due to the tiny size of them. A pair of specialized post-antennal appendages (SPAs) are only observable with the combination of CT scan and 3D rendering techniques (Figs. 2C and 2E). With the same techniques, a fractured hypostome is detected between the SPAs (Figs. 2C and 2E). Each prothoracic segment carries a small pair of biramous appendages (Figs. 2B and 2C). Four pairs of biramous appendages are found associated with every opisthothoracic segment (Figs. 2A, 2B and 2D). The biramous appendages gradually decrease in size towards the posterior of the body that terminates into a four-segmented abdomen without any appendages (T9–T12; Figs. 1 and 2B). All biramous appendages are composed of a flap-like exopod and an endopod bearing at least ten podomeres. Each podomere bears an endite medially (Figs. 2B, 2D and 2G). Similar to *Guangweicaris* (Chen et al., 2020), *th*e distal podomere of the endopod in *P. subconigera* is in subtriangular shape (Red triangle indicated in Fig. 2D). This is different from the claw-like and the rounded distal podomere seen in *Alacaris* (Yang et al., 2018) and *F. protensa* (Fu et al., 2018), respectively.

## Discussion

### Morphological comparisons among fuxianhuiids and taxonomic validity of *Pisinnocaris subconigera*

Since the original report of *Pisinnocaris subconigera* in 1998, only a few additional specimens have been assigned or considered to be related to this species due to the rarity and poor preservation of the specimens (Xu, 2004; Zeng et al., 2014). We hypothesize that the similarities shared by different members of fuxianhuiids might have led to misidentify *Pisinnocaris* as a juvenile stage of *Fuxianhuia protensa* (Fu et al., 2018).

While sharing common features with other fuxianhuiids, *Pisinnocaris subconigera* also bears unique morphological characters to support its taxonomic validity (Table 1). According to our study, the most diagnostic feature for *P. subconigera* is considered to be the prothorax composed of four shortened segments instead of three. The number of prothoracic segments is key to identify fuxianhuiids at the genus or even family levels. Notably, the tapering body shape of *P. subconigera* is similar to *Chengjiangocaris* and *Liangwangshania*, but different from *Fuxianhuia* and *Guangweicaris* whose abdomen is much narrower than the opisthothorax. From the tapering shape of the posterior segments in *P. subconigera*, one would assume those segments to be limb-bearing ones and, hence, represent part of the opisthothorax. Computed tomography (CT) scans, however, reveal no limbs for any of those segments, suggesting an abdominal identity for this tagma (Fig. 2B). Therefore, shape of the posterior segments (abrupt narrowing *vs* tapering) alone cannot serve as an indicator for the presence/absence of an abdomen in fuxianhuiids.

**Table 1:** Morphological comparisons among fuxianhuiids.

	Species								
Characters	*Liangwangshania biloba*	*Alacaris mirabilis*	*Chengjiangocaris*		*Pisinnocaris subconigera*	*Xiaocaris luoi*	*Fuxianhuia*		*Guangweicaris spinatus*
			*C. kunmingensis*	*C. longiformis*			*F. protensa*	*F. xiaoshibaensis*	
Number of trunk segments	42	13	26	26	12	15	≈ 34	≈ 37	15
Number of prothoracic segments	6	5	5	5	4	3	3	3	3
Number of opisthothoracic segments	29	5	?	?	4	6	14–19	12–18	5
Number of abdominal segments	7	3	?	?	4	6	11–14	13–17	7
Abdomen much narrower than thorax	Absent	Absent	Absent	Absent	Absent	Absent	Present	Present	Present
Shape of lateral processes	Flap-like	Flap-like	Leaf-like	Leaf-like	Leaf-like	Flap-like	Leaf-like or spine-like	Leaf-like	Leaf-like
Shape of hypostome	Inverted trapezoid	Tripartite	Tripartite	Tripartite	Inverted trapezoid	Tripartite	Inverted trapezoid	Inverted trapezoid	Tripartite
SPAs	Present	Present	Present	Present	Present	Present	Present	Present	Present
Number of appendages corresponding to each opisthothoracic segment	?	4	4	4	4	4	2	?	2
Chelate (or sub-chelate) distal podomeres	?	Present	Present	Present	Present	Present	Absent	Absent	Present
Number of endopod podomeres in trunk appendages	?	16	16	≥18	≥10	16	≈ 20	≥18	≥11
Endites on endopod	?	Present	Present	Present	Present	Present	Absent	Absent	Present

### Phylogeny

In order to clarify the phylogenetic position of *Pisinocaris subconigera*, we also performed matrix-based cladistic analysis. Both the trees from Maximum Parsimony (Figs. 3A and B) and Bayesian Inference (Fig. 3C) show that phylogenetic position of *P. subconiger* is consistent—inside the clade of fuxianhuiids. The interrelationship of different members of fuxianhuiids shown in the tree of implied weights is consistent with the Bayesian tree (Figs. 3A and 3C): among the nine fuxianhuiid members, *Liangwangshania biloba* is the sister group to all other fuxianhuiids; *A. mirabilis*, *C. longiformis* and *C. kunmingensis* cluster into a branch and *F. protensa*, *F. xiaoshibaensis*, *X. luoi* and *G. spinatus* cluster into another branch on the trees. *P. subconigera* exhibits a closer phylogenetic affinity to Fuxianhuiidae (Hou & Bergström, 1997) on the tree of implied weights. However, it is positioned closer to the stem lineage within the clade of fuxianhuiid on the Bayesian tree. The interrelationship of fuxianhuiids is less well-resolved in the tree of equal weights. The unclear details of the morphology, especially in the appendages of several other fuxianhuiids may be the reasons for this discrepancy. But this tree still supports that *P. subconigera* is a member of fuxianhuiids. By analyzing the characteristics at several nodes, it can be seen that the number of prothoracic segments plays a crucial role in classification. It is not only the most characteristic feature for fuxianhuiids as a whole being set apart from all the other Cambrian euarthropods, but also the feature that divides fuxianhuiids into four major groups (Table 1). Namely, there are six prothoracic segments in *Liangwangshania*, five in Chengjiangocarididae (Hou & Bergström, 1997), four in *P. subconigera*, and three in Fuxianhuiidae, suggesting may be four families in fuxianhuiids which currently include nine species in seven genera.

### Larval/juvenile forms

All specimens of *Pisinocaris subconigera* studied here have the same conical body shape and trunk segment number throughout the entire body and also within each tagma, despite the difference in their body size (Fig. S1). Combined with the discovery of the largest individual of *P. subconigera* with a total body length of 47.62 mm and the prothoracic segments and appendage morphology of other individuals revealed by micro-CT, we conclude that *P. subconigera* is not the larval or juvenile form of any other fuxianhuiids, but is a valid species on its own.

**Figure 4:**
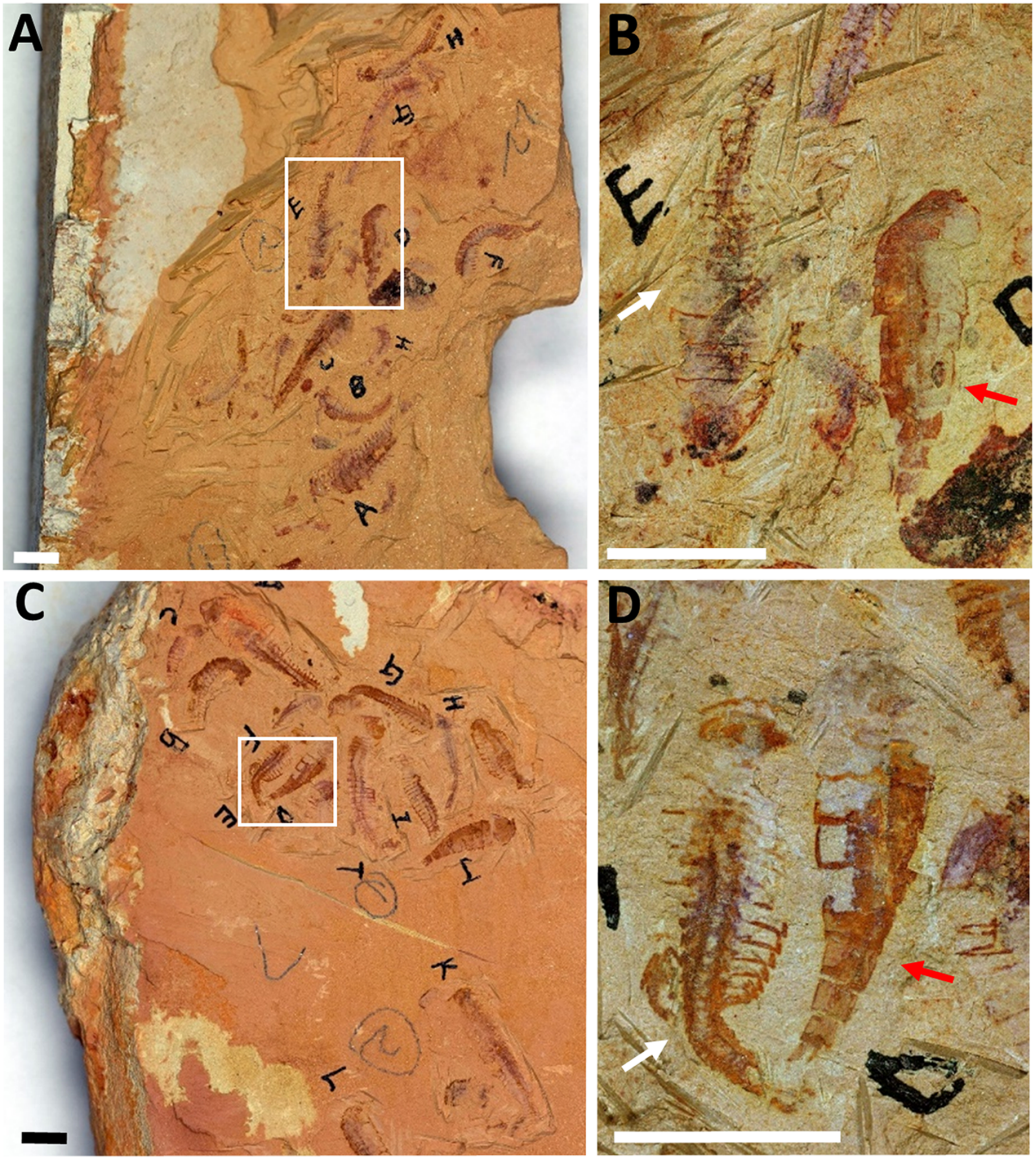
*Pisinnocaris subconigera* and *Fuxianhuia protensa* preserved together. (A) YRCP-R-0001, from Maotianshan, Chengjiang, showing several small *F. protensa* and *P. subconigera* were preserved together. (B) The white-framed part in A, showing a *F. protensa* (left one marked with white arrow) with about twenty-four trunk segments and a *P. subconigera* (right one marked with red arrow indicator) of eight trunk segments. The white arrow shows the smallest individual with at least twelve segments. (C) YRCP-R-0024, from Maotianshan, Chengjiang, showing several small *F. protensa* and *P. subconigera* were preserved together. (D) is the white-framed part in C, showing a *F. protensa* (left one marked with white arrow) with about twenty-five trunk segments and a *P. subconigera* (right one marked with red arrow) with eight trunk segments. Scale bars: 5 mm.

Another reason for considering *P. subconigera* a synonym of juvenile *F. protensa* is the co-occurance of *P. subconigera* and an adult of *F. protensa* on the same fossil slab (fig. 13 in Fu et al., 2018). Similar slabs are also shown here, on which *P. subconigera* and the juvenile of *F. protensa* (maybe stage 24 or 25, Figs. 4B and 4D) have similar body size, but differences in body shape and trunk segmentation between the two species are clearly observable (Fig. 4). As a consequence, it remains an open question whether extended parental care exists in *F. protensa*. We cannot rule out the possibility that they may simply be preserved together. More specimens of *F. protensa* with different developmental stages are key to clarifying the situation.

**Figure 5:**
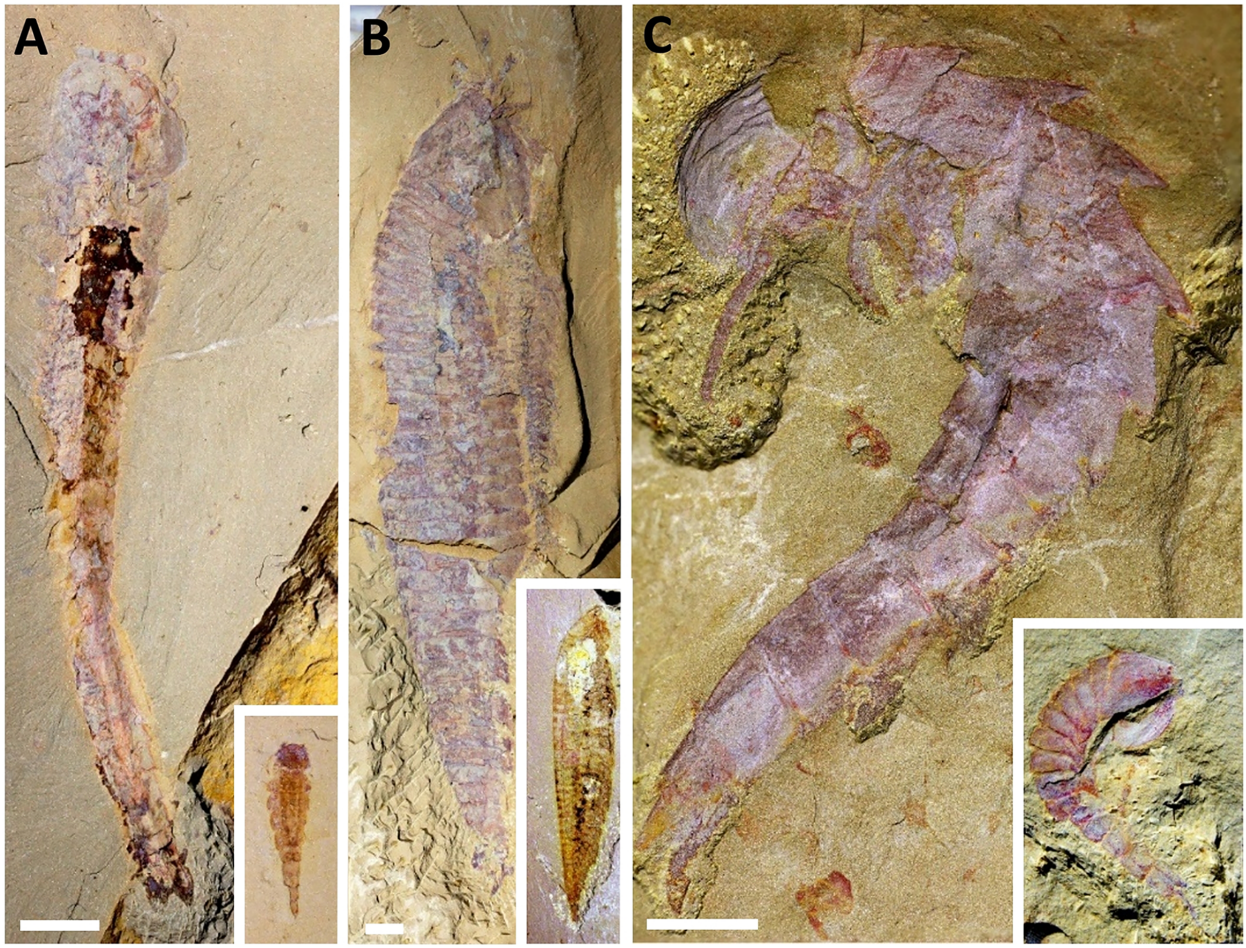
Adult and presumed larval/juvenile specimens of other fuxianhuiids. (A) *Chengjiangocaris longiformis* (YKLP17302) and a presumed larva/juvenile (YKLP17303). (B) *Liangwangshania biloba* (YKLP17304) and a presumed larva/juvenile (YKLP17305). (C) *Guangweicaris spinatus* (YKLP11566-arthro) and a presumed larva/juvenile (YKLP17306). Scale bars: 5 mm.

In fossil research, it is extremely difficult to accurately identify the larval/juvenile and adult forms due to the limited number of specimens and the preservation. The specimens of the same species often vary greatly in size, leading to predictions about larval, juvenile or adult forms. In addition to appearance and preservation of fossils, more details of morphological characteristics need to be considered, especially when conducting studies on ontogeny, such as changing trunk segmentation and morphological features of appendages (Fu et al., 2018; Liu et al., 2020b and Zhai et al., 2019). The same situation exists in fuxianhuiids. Small individual specimens have been found and they were presumed to the larval or juvenile form of fuxianhuiids (Fig. 5) often based on being highly similar in appearance. For example, in Fig. 5A, the large fossil individual has been identified as *C. longiformis*, and the small one exhibits identical morphological traits, including its long and narrow body, carapace shape, and tail structure. *L. biloba*, with the small individual specimens shown in Fig. 5B and *G. spinatus* with the small individual specimens shown in Fig. 5C display similar phenomena. In fact, we need more evidence, especially details of appendage morphology, to identify the species of these three small individuals and determine whether they are larve or juveniles, just as we did with *P. subconigera* in this study.

## Conclusions

- *Pisinnocaris subconigera* is a valid species of early Cambrian fuxianhuiid, not a larval/juvenile form of any other fuxianhuiid including *Fuxianhuia protensa*.
- Study of larval/juvenile forms of fossil species requires not only more specimens from various developmental stages but also consideration of more details of morphological characteristics.

## Supplemental Information

10.7717/peerj.20483/supp-1 Supplemental Information 1 Supplementary Materials.

10.7717/peerj.20483/supp-2 Supplemental Information 2 Different size of *P. subconigera* individual. (A) A total of fourteen individuals are arranged from left to right according to their sizes. The specimen numbers are, in order: YRCP-R-0013-D, YRCP-R-0027-J, YRCP-R-0024-D, NIGPAS 115417a, YRCP-R-0007-A, YRCP-R-0024-J, YRCP-R-0001-D, YRCP-R-0020-F, YRCP-R-0019-D, YRCP-R-0036b, CJHMD00070, YRCP-R-0034, YKLP17301 and CJHMD00066a. (B) Scatterplot of the fourteen individuals, X axis represents the body length and Y axis represents the width of T5, T5 bears the widest tergite throughout the entire body. Trend line with blue dotted suggested that length-to-width ratio of different sizes individuals tends to be consistent.

10.7717/peerj.20483/supp-3 Supplemental Information 3 Distribution of the main localities of the Chengjiang biota, Yunnan Province and the stratum. (A) Red box shows the “Xiaolantian” section. (B) Stratigraphic column of the main localities of the Chengjiang biota (revised from Hou et al., 2017 and Jin et al., 2024)

10.7717/peerj.20483/supp-4 Supplemental Information 4 Information of the specimens analyzed in the present study.

10.7717/peerj.20483/supp-5 Supplemental Information 5 Characters list.

10.7717/peerj.20483/supp-6 Supplemental Information 6 Matrix for TNT.

10.7717/peerj.20483/supp-7 Supplemental Information 7 Matrix for Mrbayes.

## Additional Information and Declarations

### Data Availability

The following information was supplied regarding data availability:

The computed tomography data is available at Mendeley Data: Mai, Huijuan; Chen, Hong; Guo, Jin; Chen, Ailin; Hou, Xianguang; Liu, Yu (2025), “3D Dataset of the Cambrian arthropod fossil Pisinnocaris subconigera from Chengjiang biota”, Mendeley Data, V2, doi: 10.17632/z5dvmvkfjy.2.

## References

[ref-1] Aria C, Zhao F, Zhu M (2021). Fuxianhuiids are mandibulates and share affinities with total-group Myriapoda. Journal of the Geological Society.

[ref-2] Chen A (2005). A new Fuxianhuia-like arthropod of the early Cambrian Chengjiang fauna in Yunnan. Yunnan Geology.

[ref-3] Chen H (2020). Restudy of the fuxianhuiid arthropods from Cambrian Series 2, Eastern Yunnan. Ph.D. dissertation, Yunnan University, Tunnan, China (in Chinese with English abstract).

[ref-4] Chen A, Chen H, Legg DA, Liu Y, Hou X (2018). A redescription of *Liangwangshania biloba* Chen, 2005, from the Chengjiang biota (Cambrian, China), with a discussion of possible sexual dimorphism in fuxianhuiid arthropods. Arthropod Structure & Development.

[ref-5] Chen H, Legg DA, Zhai D, Liu Y, Hou X (2020). New data on the anatomy of fuxianhuiid arthropod Guangweicaris spinatus from the lower Cambrian Guanshan Biota, Yunnan, China. Acta Palaeontologica Polonica.

[ref-6] Du K, Ortega-Hernández J, Yang J, Zhang X (2019). A soft-bodied euarthropod from the early Cambrian Xiaoshiba Lagerstätte of China supports a new clade of basal artiopodans with dorsal ecdysial sutures. Cladistics.

[ref-7] Fu D, Ortega-Hernández J, Daley AC, Zhang X, Shu D (2018). Anamorphic development and extended parental care in a 520 million-year-old stem-group euarthropod from China. BMC Evolutionary Biology.

[ref-8] Gabbott SE, Hou X, Norry MJ, Siveter DJ (2004). Preservation of early Cambrian animals of the Chengjiang biota. Geology.

[ref-9] Hou X (1987). Three new large arthropods from lower Cambrian, Chengjiang, eastern Yunnan. Acta Palaeontologica Sinica.

[ref-10] Hou X, Bergström J, Wang H, Feng X, Chen A (1999). The Chengjiang fauna- exceptionally well-preserved animals from 530 million years ago.

[ref-11] Hou X, Bergström J (1991). The arthropods of the Lower Cambrian Chengjiang fauna, with relationships and evolutionary significance. The Early Evolution of Metazoa and the Significance of Problematic Taxa.

[ref-12] Hou X, Bergström J (1997). Arthropods of the lower Cambrian Chengjiang fauna, southwest China. Fossils and Strata.

[ref-13] Hou X, Bergström J (1998). Three additional arthropods from the early Cambrian Chengjiang fauna, Yunnan, southwest China. Acta Palaeontologica Sinica.

[ref-14] Hou X, Siveter DJ, Siveter DJ, Aldridge RJ, Cong P, Gabbott SE, Ma X, Purnell MA, Williams M (2017). The Cambrian Fossils of Chengjiang, China (the flowering of early animal Life) || Algae.

[ref-15] Liu Y, Ortega-Hernández J, Chen H, Mai H, Zhai D, Hou X (2020a). Computed tomography sheds new light on the affinities of the enigmatic euarthropod *Jianshania furcatus* from the early Cambrian Chengjiang biota. BMC Evolutionary Biology.

[ref-16] Liu Y, Ortega-Hernández J, Zhai D, Hou X (2020b). A reduced labrum in a Cambrian great-appendage euarthropod. Current Biology.

[ref-17] Luo H, Fu X, Hu S, Li Y, Hou S, You T, Pang J, Liu Q (2007). A new arthropod, *Guangweicaris* Luo, Fu et Hu gen. nov. from the Early Cambrian Guanshan Fauna, Kunming. China Acta Geologica Sinica.

[ref-18] Ortega-Hernández J, Yang J, Zhang X (2018). Fuxianhuiids. Current Biology.

[ref-19] Ronquist F, Teslenko M, Van Der Mark P, Ayres DL, Darling A, Höhna S, Larget B, Liu L, Suchard MA, Huelsenbeck JP (2012). MrBayes 3.2: efficient Bayesian phylogeneticinference and model choice across a large model space. Systematic Biology.

[ref-20] Waloszek D, Chen J, Maas A, Wang X (2005). Early Cambrian arthropods—new insights into arthropod head and structural evolution. Arthropod Structure & Development.

[ref-22] Xu G (2004). New specimens of rare arthropods from the early Cambrain Chengjiang fauna, Yunnan, China. Acta Palaeontologica Sinica.

[ref-23] Yang J, Ortega-Hernández J, Butterfield NJ, Zhang X (2013). Specialized appendages in fuxianhuiids and the head organization of early euarthropods. Nature.

[ref-24] Yang J, Ortega-Hernández J, Legg DA, Lan T, Hou J, Zhang X (2018). Early Cambrian fuxianhuiids from China reveal origin of the gnathobasic protopodite in euarthropods. Nature Communications.

[ref-25] Zeng H, Zhao F, Yin Z, Li G, Zhu M (2014). A Chengjiang-type fossil assemblage from the Hongjingshao Formation (Cambrian Stage 3) at Chenggong, Kunming. Yunnan Chinese Science Bulletin.

[ref-26] Zhai D, Edgecombe GD, Bond AD, Mai H, Hou X, Liu Y (2019). Fine-scale appendage structure of the Cambrian trilobitomorph *Naraoia spinosa* and its ontogenetic and ecological implications. Proceedings of the Royal Society B: Biological Sciences.

[ref-27] Zhang X, Liu Y, O’Flynn RJ, Schmidt M, Melzer RR, Hou X, Mai H, Guo J, Yu M, Ortega-Hernández J (2022). Ventral organization of *Jianfengia multisegmentalis* Hou, and its implications for the head segmentation of megacheirans. Palaeontology.

