# Supplementary material for: Pisinnocaris subconigera—a valid species of early Cambrian fuxianhuiid
Source: PeerJ. 2026 Feb 3;14:e20483. doi: 10.7717/peerj.20483 (PMC12880106; doi:10.7717/peerj.20483)
Supplement: Supplemental Information 1 [file peerj-14-20483-s001.docx]

Supplementary Information for

***Pisinnocaris subconigera* — a valid species of early Cambrian fuxianhuiid**

Huijuan Mai^1,2,^*, Hong Chen^3,4,^*, Ailin Chen^5^, Jin Guo^1,2,6^, Xianguang Hou^1,2^，Yu Liu^1,2,6,7,8^

^1^Yunnan Key Laboratory for Palaeobiology, Institute of Palaeontology, Yunnan University, Kunming, Yunnan, China

^2^MEC International Joint Laboratory for Palaeobiology and Palaeoenvironment, Yunnan University, Kunming, Yunnan, China

^3^School of Biological Sciences and Technology, Liupanshui Normal University, Liupanshui, Guizhou, China

^4^Guizhou Provincial Key Laboratory for Palaeontology and Palaeoenvironment, Guizhou University, Guiyang, Guizhou, China

^5^Research Center of Paleobiology, Yuxi Normal University, Yuxi, Yunnan, China

^6^Chengjiang Fossil Museum of the Management Committee of the Chengjiang World Heritage Fossil Site, Chengjiang, Yunnan, China

^7^Southwest United Graduate School, Kunming, Yunnan, China

^8^School of Geography, Geology and the Environment, University of Leicester, University Road, Leicester LE1 7RH, UK

*These authors contributed equally

Corresponding Author: Xianguang Hou and Yu Liu

South Waihuan Road, Chenggong District, Kunming, Yunnan, 650500, China

Email address: Xianguang Hou, xghou@ynu.edu.cn; Yu Liu, yu.liu@ynu.edu.cn

**Supplementary Informaion includes:**

Figs. S1 to S2

Tables S1

Data S1: Characters list (separate file)

Data S2: Matrix for TNT (separate file)

Data S3: Matrix for Mrbayes (separate file)


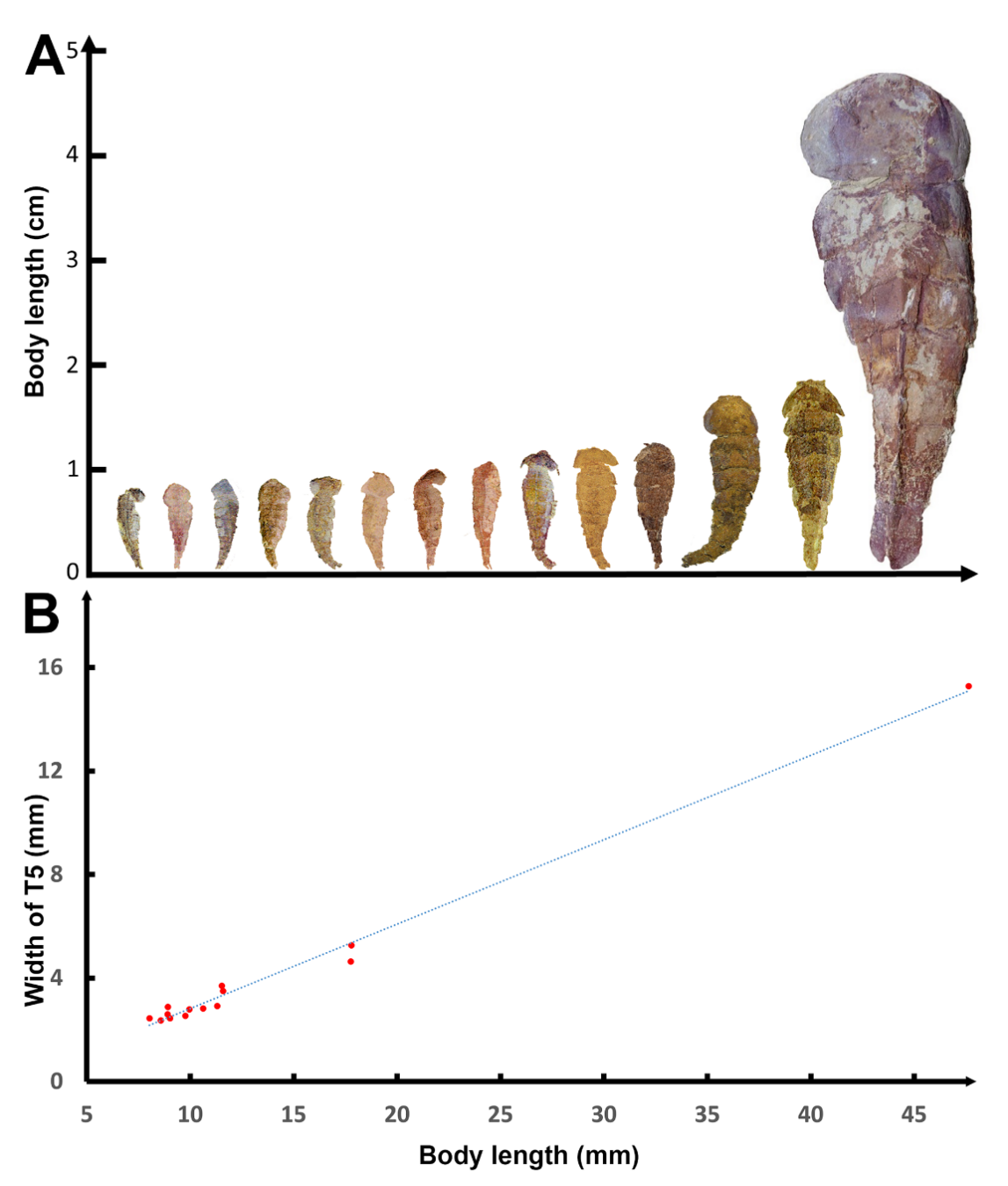


**Figure S1. Different size of *P. subconigera* individual.** (A) A total of fourteen individuals are arranged from left to right according to their sizes. The specimen numbers are, in order: YRCP-R-0013-D, YRCP-R-0027-J, YRCP-R-0024-D, NIGPAS 115417a, YRCP-R-0007-A, YRCP-R-0024-J, YRCP-R-0001-D, YRCP-R-0020-F, YRCP-R-0019-D, YRCP-R-0036b, CJHMD00070, YRCP-R-0034, YKLP17301 and CJHMD00066a. (B) Scatterplot of the fourteen individuals, X axis represents the body length and Y axis represents the width of T5, T5 bears the widest tergite throughout the entire body. Trend line with blue dotted suggested that length-to-width ratio of different sizes individuals tends to be consistent.


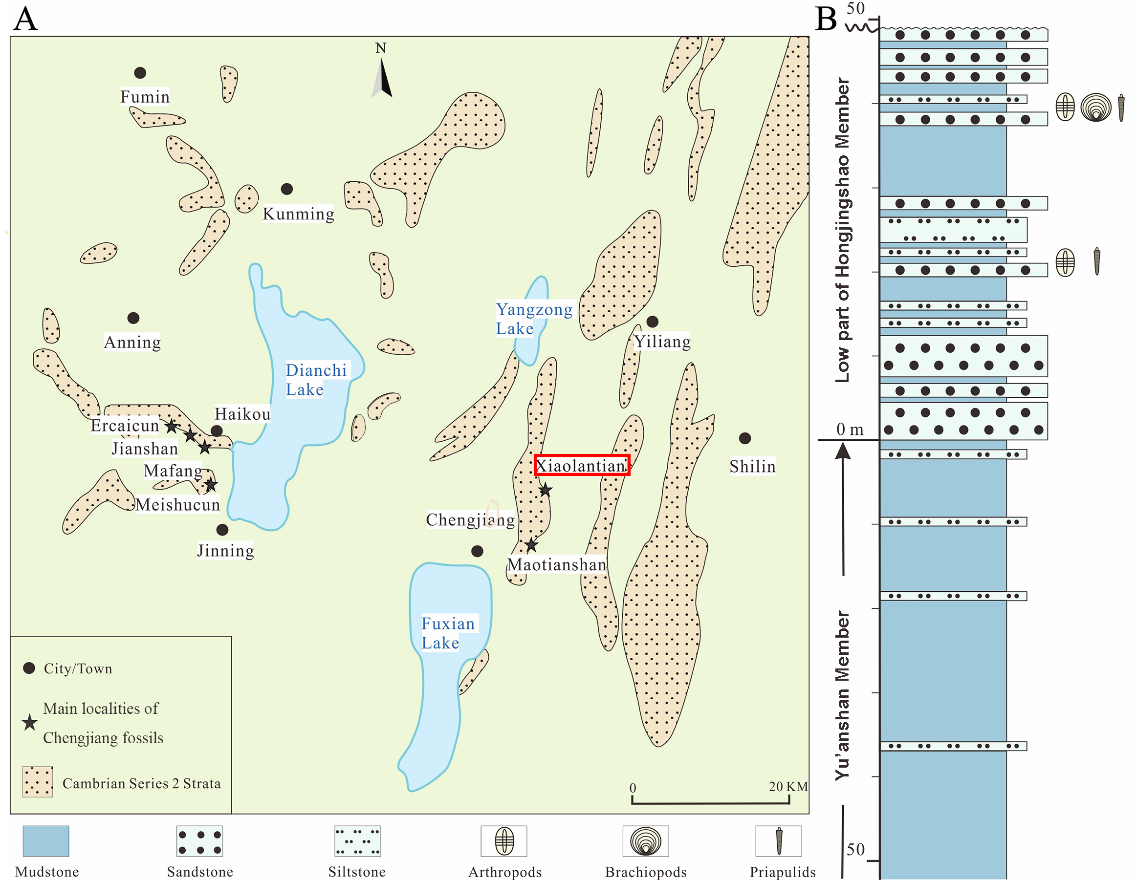


**Figure S2. Distribution of the main localities of the Chengjiang biota, Yunnan Province and the stratum.** (A) Red box shows the “Xiaolantian” section. (B) Stratigraphic column of the main localities of the Chengjiang biota (revised from *Hou et al, 2017 and Jin et al, 2024*)

Table S1. Information of the specimens analyzed in the present study.

| **Specimen** | **Horizon** | **Body length (mm)** | **Width of T5**  **(mm)** | **Preservation** | **deposited** | **Figures** |
| --- | --- | --- | --- | --- | --- | --- |
| NIGPAS 115417a | Yu’anshan Member | 8.92 | 2.89 | Whole body | Yunnan Key Laboratory for Palaeobiology | Figs. 1A, 2A and S1A |
| CJHMD00070 | Yu’anshan Member | 11.58 | 3.51 | Whole body | Chengjiang Fossil Museum | Figs. 1B and S1 |
| YKLP17301 | Yu’anshan Member | 17.79 | 5.27 | Whole body | Yunnan Key Laboratory for Palaeobiology | Figs. 1C, 2B, C, D and S1 |
| CJHMD00066a | Yu’anshan Member | 47.62 | 15.28 | Whole body | Chengjiang Fossil Museum | Figs. 1D, 2E and S1 |
| YRCP-R-0013-D | Yu’anshan Member | 8.02 | 2.45 | Whole body | Yuxi Normal University | Fig. S1 |
| YRCP-R-0027-J | Yu’anshan Member | 8.58 | 2.37 | Whole body | Yuxi Normal University | Fig. S1 |
| YRCP-R-0024-D | Yu’anshan Member | 8.90 | 2.60 | Whole body | Yuxi Normal University | Fig. S1 |
| YRCP-R-0007-A | Yu’anshan Member | 9.02 | 2.45 | Whole body | Yuxi Normal University | Fig. S1 |
| YRCP-R-0024-J | Yu’anshan Member | 9.76 | 2.54 | Whole body | Yuxi Normal University | Fig. S1 |
| YRCP-R-0001-D | Yu’anshan Member | 9.95 | 2.79 | Whole body | Yuxi Normal University | Fig. S1 |
| YRCP-R-0020-F | Yu’anshan Member | 10.61 | 2.83 | Whole body | Yuxi Normal University | Fig. S1 |
| YRCP-R-0019-D | Yu’anshan Member | 11.30 | 2.92 | Whole body | Yuxi Normal University | Fig. S1 |
| YRCP-R-0036b | Yu’anshan Member | 11.52 | 3.71 | Whole body | Yuxi Normal University | Fig. S1 |
| YRCP-R-0034 | Yu’anshan Member | 17.76 | 4.64 | Whole body | Yuxi Normal University | Fig. S1 |
| YRCP-R-0001 | Yu’anshan Member | - | - | Slab | Yuxi Normal University | Fig. 4A and B |
| YRCP-R-0024 | Yu’anshan Member | - | - | Slab | Yuxi Normal University | Fig. 4C and D |
| YKLP17302 | Yu’anshan Member | - | - | Whole body | Yunnan Key Laboratory for Palaeobiology | Fig. 5A |
| YKLP17303 | Yu’anshan Member | - | - | Whole body | Yunnan Key Laboratory for Palaeobiology | Fig. 5A |
| YKLP17304 | Yu’anshan Member | - | - | Whole body | Yunnan Key Laboratory for Palaeobiology | Fig. 5B |
| YKLP17305 | Yu’anshan Member | - | - | Whole body | Yunnan Key Laboratory for Palaeobiology | Fig. 5B |
| YKLP11566-arthro | Wulongqing Member | - | - | Whole body | Yunnan Key Laboratory for Palaeobiology | Fig. 5C |
| YKLP17306 | Wulongqing Member | - | - | Whole body | Yunnan Key Laboratory for Palaeobiology | Fig. 5C |

**Reference**

Jin, C., Chen, H., Mai, H., Hou, X., Yang, X. and Zhai, D. (2024). "Discovery of diverse Pectocaris species at the Cambrian series 2 Hongjingshao formation Xiazhuang section (Kunming, SW China) and its ecological, taphonomic, and biostratigraphic implications." *PeerJ* 12.

Hou, X., Siveter D J., Siveter D J., Aldridge, R J., Cong, P., Gabbott, S E., Ma, X., MA Purnell, M A. and Williams, M. (2017). The Cambrian Fossils of Chengjiang, China (The Flowering of Early Animal Life) || Algae. John Wiley & Sons Press.
