# Supplementary figures and images for: Pisinnocaris subconigera—a valid species of early Cambrian fuxianhuiid
Source: PeerJ. 2026 Feb 3;14:e20483. doi: 10.7717/peerj.20483 (PMC12880106; doi:10.7717/peerj.20483)

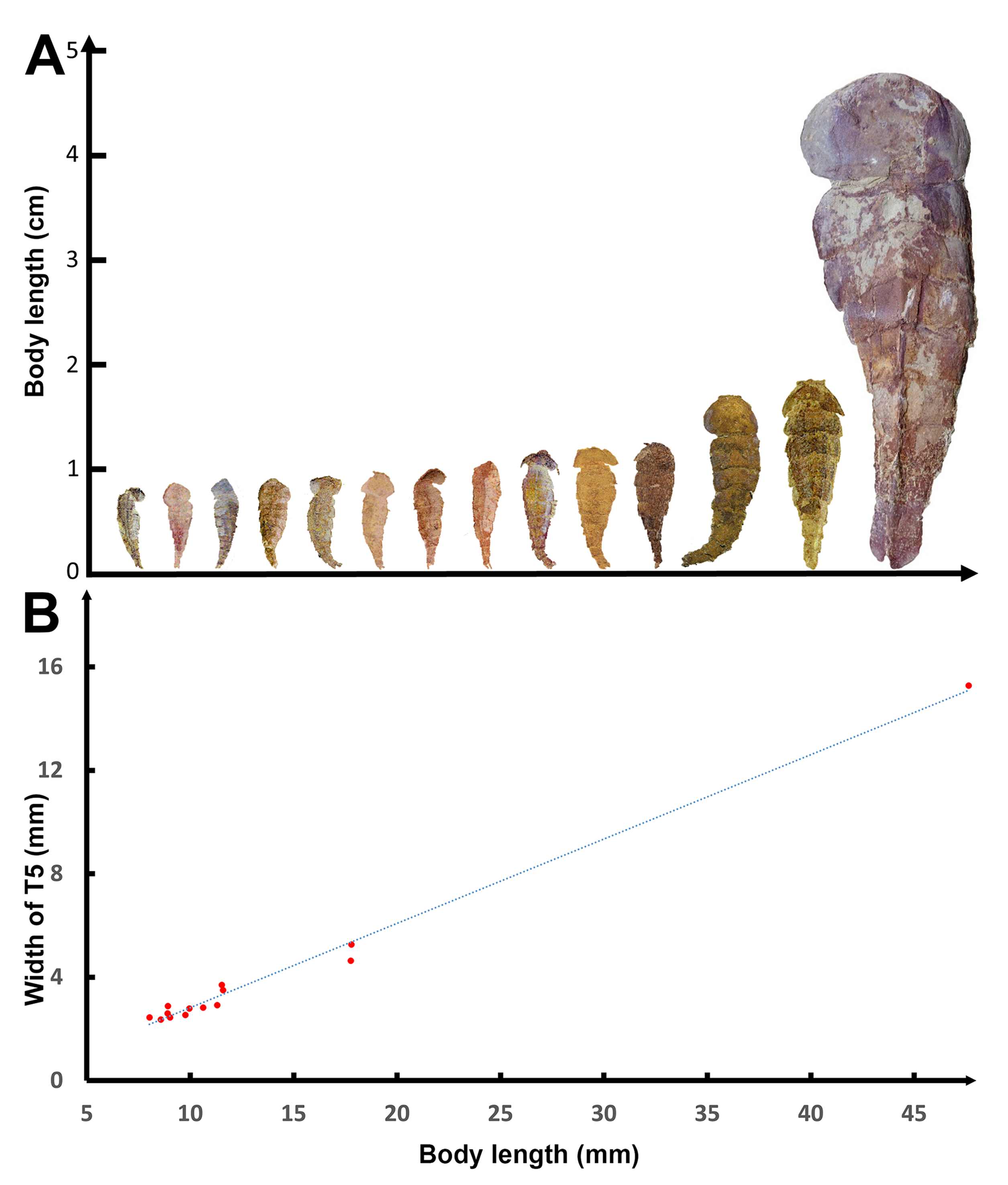

Supplement: Supplemental Information 2 — (A) A total of fourteen individuals are arranged from left to right according to their sizes. The specimen numbers are, in order: YRCP-R-0013-D, YRCP-R-0027-J, YRCP-R-0024-D, NIGPAS 115417a, YRCP-R-0007-A, YRCP-R-0024-J, YRCP-R-0001-D, YRCP-R-0020-F, YRCP-R-0019-D, YRCP-R-0036b, CJHMD00070, YRCP-R-0034, YKLP17301 and CJHMD00066a. (B) Scatterplot of the fourteen individuals, X axis represents the body length and Y axis represents the width of T5, T5 bears the widest tergite throughout the entire body. Trend line with blue dotted suggested that length-to-width ratio of different sizes individuals tends to be consistent. [file peerj-14-20483-s002.png]

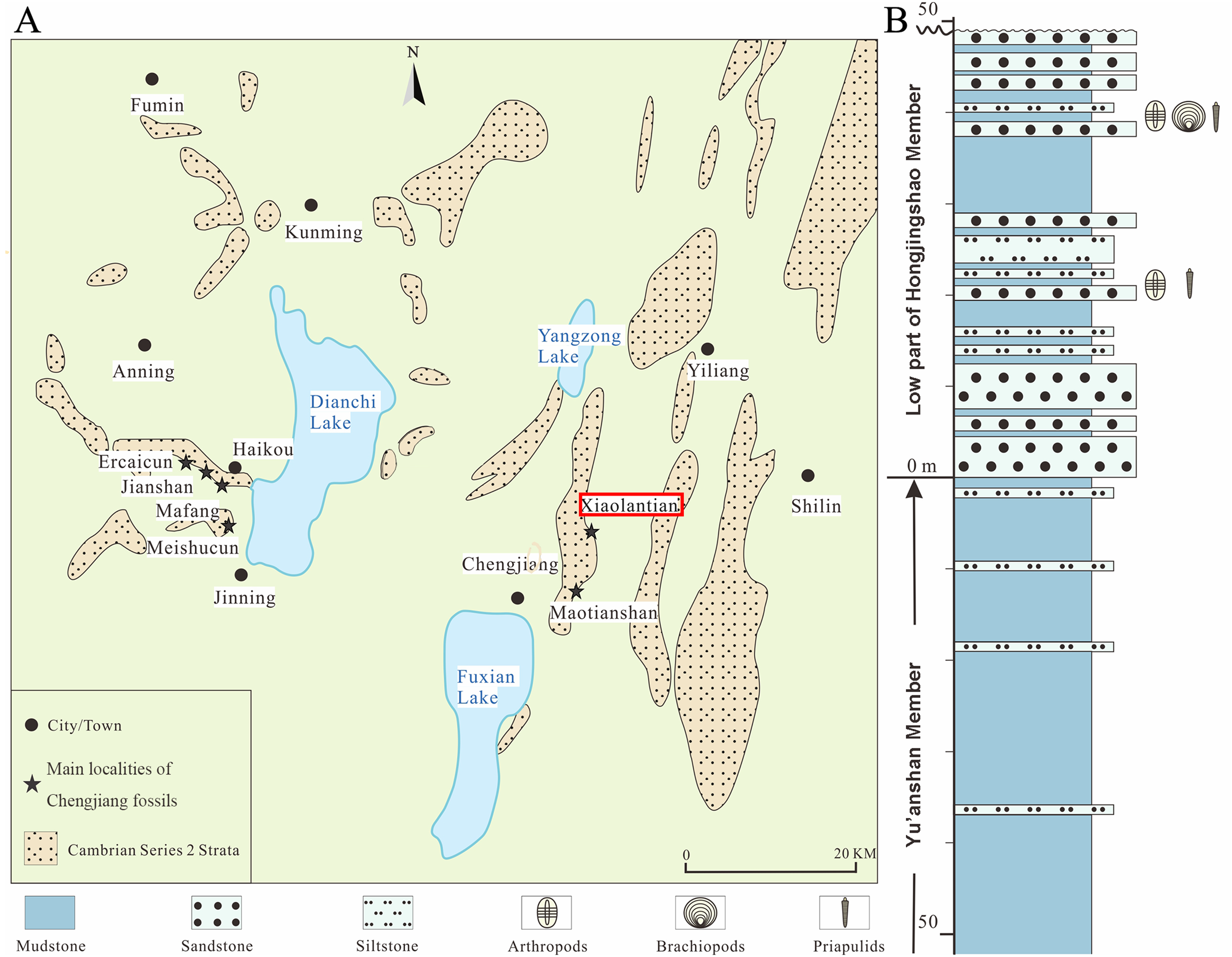

Supplement: Supplemental Information 3 — (A) Red box shows the “Xiaolantian” section. (B) Stratigraphic column of the main localities of the Chengjiang biota (revised from Hou et al., 2017 and Jin et al., 2024) [file peerj-14-20483-s003.png]
