# Supplementary material for: Pisinnocaris subconigera—a valid species of early Cambrian fuxianhuiid
Source: PeerJ. 2026 Feb 3;14:e20483. doi: 10.7717/peerj.20483 (PMC12880106; doi:10.7717/peerj.20483)
Supplement: Supplemental Information 4 [file peerj-14-20483-s004.docx]

Table S1. Information of the specimens analyzed in the present study.

| **Specimen** | **Horizon** | **Body length (mm)** | **Width of T5**  **(mm)** | **Preservation** | **deposited** | **Figures** |
| --- | --- | --- | --- | --- | --- | --- |
| NIGPAS 115417a | Yu’anshan Member | 8.92 | 2.89 | Whole body | Yunnan Key Laboratory for Palaeobiology | Figs. 1A, 2A and S1A |
| CJHMD00070 | Yu’anshan Member | 11.58 | 3.51 | Whole body | Chengjiang Fossil Museum | Figs. 1B and S1 |
| YKLP17301 | Yu’anshan Member | 17.79 | 5.27 | Whole body | Yunnan Key Laboratory for Palaeobiology | Figs. 1C, 2B, C, D and S1 |
| CJHMD00066a | Yu’anshan Member | 47.62 | 15.28 | Whole body | Chengjiang Fossil Museum | Figs. 1D, 2E and S1 |
| YRCP-R-0013-D | Yu’anshan Member | 8.02 | 2.45 | Whole body | Yuxi Normal University | Fig. S1 |
| YRCP-R-0027-J | Yu’anshan Member | 8.58 | 2.37 | Whole body | Yuxi Normal University | Fig. S1 |
| YRCP-R-0024-D | Yu’anshan Member | 8.90 | 2.60 | Whole body | Yuxi Normal University | Fig. S1 |
| YRCP-R-0007-A | Yu’anshan Member | 9.02 | 2.45 | Whole body | Yuxi Normal University | Fig. S1 |
| YRCP-R-0024-J | Yu’anshan Member | 9.76 | 2.54 | Whole body | Yuxi Normal University | Fig. S1 |
| YRCP-R-0001-D | Yu’anshan Member | 9.95 | 2.79 | Whole body | Yuxi Normal University | Fig. S1 |
| YRCP-R-0020-F | Yu’anshan Member | 10.61 | 2.83 | Whole body | Yuxi Normal University | Fig. S1 |
| YRCP-R-0019-D | Yu’anshan Member | 11.30 | 2.92 | Whole body | Yuxi Normal University | Fig. S1 |
| YRCP-R-0036b | Yu’anshan Member | 11.52 | 3.71 | Whole body | Yuxi Normal University | Fig. S1 |
| YRCP-R-0034 | Yu’anshan Member | 17.76 | 4.64 | Whole body | Yuxi Normal University | Fig. S1 |
| YRCP-R-0001 | Yu’anshan Member | - | - | Slab | Yuxi Normal University | Fig. 4A and B |
| YRCP-R-0024 | Yu’anshan Member | - | - | Slab | Yuxi Normal University | Fig. 4C and D |
| YKLP17302 | Yu’anshan Member | - | - | Whole body | Yunnan Key Laboratory for Palaeobiology | Fig. 5A |
| YKLP17303 | Yu’anshan Member | - | - | Whole body | Yunnan Key Laboratory for Palaeobiology | Fig. 5A |
| YKLP17304 | Yu’anshan Member | - | - | Whole body | Yunnan Key Laboratory for Palaeobiology | Fig. 5B |
| YKLP17305 | Yu’anshan Member | - | - | Whole body | Yunnan Key Laboratory for Palaeobiology | Fig. 5B |
| YKLP11566-arthro | Wulongqing Member | - | - | Whole body | Yunnan Key Laboratory for Palaeobiology | Fig. 5C |
| YKLP17306 | Wulongqing Member | - | - | Whole body | Yunnan Key Laboratory for Palaeobiology | Fig. 5C |
