# Supplementary material for: Pisinnocaris subconigera—a valid species of early Cambrian fuxianhuiid
Source: PeerJ. 2026 Feb 3;14:e20483. doi: 10.7717/peerj.20483 (PMC12880106; doi:10.7717/peerj.20483)
Supplement: Supplemental Information 5 [file peerj-14-20483-s005.docx]

Characters List

Continuous

*Cephalic (carapace)*

0. *Length to width ratio of anterior sclerite*.

Inapplicable for taxa lacking a distinct anterior sclerite (ch. 17). An elongate anterior sclerite, compared to width, is seen in the hurdids, specifically *Hurdia victoria* and *Aegirocassis benmoulae*, whilst the anomalocaridids, and upper-stem-group euarthropods have an anterior sclerite that is wider than long.

1. *Tallest point of carapace.*

Inapplicable for taxa lacking a carapace (ch. 24). There is a lot of variety of this character amongst “carapace-bearing arthropods”, with isoxyids typically possessing a shallow carapace posterior with the tallest point occurring in the anterior half of the domicilium, whist protocaridids are more rounded, with the tallest point occurring towards the middle, and fuxianhuids towards the posterior of the carapace.

2. *Height to length ratio of carapace domiciluium.*

Inapplicable for taxa lacking a carapace (ch. 24). This character serves to distinguish the carapaces of most “carapace-bearing arthropods”, in which the carapace is typically longer than wide, from fuxianhuids, in which the carapace is wider than long.

3. *Length of antero-dorsal carapace spines*.

Inapplicable for taxa lacking antero-dorsal carapace spines (ch. 29). This character specifically refers to species of *Isoxys*. Both *I. volucris*, and *I. curvirostratus* possess an elongate antero-dorsal spine, whilst that of *I. acutangulus* and *I. auritus* is much shorter.

4. *Length of postero-dorsal carapace spines*.

Inapplicable for taxa lacking postero-dorsal carapace spines (ch. 34). This character specifically refers to species of *Isoxys*.

5. *Slope of posterior carapace margin*.

Inapplicable for taxa lacking a carapace (ch. 24). This character serves to distinguish *Isoxys* from other species of “carapace-bearing arthropods”. *Isoxys* is unique in possessing a deeply sloped posterior carapace margin.

Meristic

Meristic characters were analysed as continuous characters. This differs from previous analyses that have treated them as discrete, thereby creating arbitrary bins and reducing character linkage and hindering trait determination.

*Trunk*

6. *Number of trunk segments*.

This character could not be determined for many taxa bearing a carapace, as this feature frequently covers an anterior series of poorly sclerotized trunk segments. Likewise, this character was not coded for the trilobite *Olenoides serratus*, as the exact number of segments in the pygidium could not be determined.

7. *Number of prothoracic segments*.

This character is inapplicable for taxa lacking a prothorax (ch. 43). The fuxianhuidids, namely *Guangweicaris spinatus*, *Xiaocaris luoi*, *Fuxianhuia protensa*, and *F. xiaoshibaensis*, possess three prothoracic segments, whilst *Pisinnocaris subconigera* possess four, and the chengjiangocarids, *Alacaris multinoda*, *Chengjiangocaris longiformis*, and *C. kunmingensis*, possess five, whilst *Liangwangshania biloba* possesses six.

8. *Number of post-prothoracic (opithothoracic) thorax segments*.

9. *Number of abdominal segments*.

This character is inapplicable for taxa lacking a distinct abdomen delineated by either a lack of appendages (ch. 44), or a notable change in dimension from the anterior (thoracic) trunk segments (ch. 45).

*Appendicular (cephalic)*

10. *Number of podomeres in pre-antennular (protocerebral) appendages*.

This character is inapplicable for taxa lacking an arthropodized protocerebral appendage with distinct podomeres (ch. 49).

11. *Number of post-protocerebral cephalic appendage pairs in cephalic region*.

This character is inapplicable for taxa lacking a distinct sclerotized cephalic covering (ch. 23), and could not be determined in most “carapace-bearing arthropods” due to uncertainty regarding the position of the cephalon-trunk boundary. In fuxianhuids this can be determined based on the position of the prothorax relative to the anterior appendages.

12. *Number of podomeres in differentiated tritocerebral appendage*.

This character is inapplicable for taxa lacking a pair of differentiated tritocerebral appendage (ch. 62). All fuxianhuids for which a differentiated tritocerebral appendage is known possess three segments in each appendage, whilst megacheirans possess either five or six, and the protocaridids, *Branchiocaris pretiosa* and *Tokumnia katalepis* both possess eight.

13. *Number of spine-bearing distal podomeres on tritocerebral appendage.*

This character is inapplicable for taxa lacking spinose projections on a differentiated tritocerebral appendage (ch. 65).

*Appendicular (general)*

14. *Number of exite segments*.

This character is inapplicable for taxa lacking exites (ch. 66). This character serves to distinguish advanced megacheirans, specifically *Yohoia tenuis* and *Leanchoilia superlata*, and the trilobite *Olenoides serratus*, which possess an exite composed of two segments, from the other megacheirans, and stem-lineage euarthropods, including the dinocaridids, and “carapace-bearing arthropods”, which possess just a singular segment.

15. *Number of trunk endopod segments (podomeres).*

*Appendicular (lateral processes)*

16. *Number of lateral process pairs*.

Discrete

*Cephalic (including external ocular features)*

17. *Anterior (ocular) sclerite: (0) absent, (1) present*.

18. *Anterior margin of anterior sclerite tapered: (0) absent, (1) present*.

19. *Anterior sclerite flanked by lateral plates: (0) absent, (1) present*.

20. *Lateral eyes: (0) absent, (1) present*.

21. *Lateral eyes stalked: (0) absent, (1) present*.

22. *Single medial eye: (0) absent, (1) present*.

23. *Sclerotized cephalic covering: (0) absent, (1) present*.

*Cephalic (carapace)*

24. *Free posterolateral extensions of sclerotized cephalic covering: (0) absent, (t) present*.

25. *Antero-ventral and postero-dorsal margins of carapace parallel: (0) absent, (1) present*.

26. *Carapace reticulate: (0) absent, (1) present*.

27. *Reticulation type: (0) small mesh, (1) large, hexagonal, mesh*.

28. *Anterior extension of carapace: (0) absent, (1) present*.

29. *Anterodorsal spine: (0) absent, (1) present*.

30. *Anteroventral hook-like processes: (0) absent, (1) present*.

31. *Posterodorsal keel: (0) absent, (1) present*.

32. *Carapace divided medial by suture or hinge: (0) absent, (1) present*.

33. *Posteromedial suture on carapace: (0) absent, (1) present*.

34. *Posterodorsal spine: (0) absent, (1) present*.

35. *Lateral margins of carapace contiguous with lateral pleural trunk margins: (0) absent, (1) present.*

*Trunk*

36. *Sclerotized trunk: (0) absent, (1) present*.

37. *Paired dorsal nodes: (0) absent, (1) present*.

38. *Axial carinae: (0) absent, (1) present*.

39. *Dorsal band of blade-like setae: (0) absent, (1) present*.

40. *Pleural extension of tergites: (0) absent, (1) present*.

41. *Posterolateral margins of pleurae extended into spinose projections: (0) absent, (1) present*.

42. *Raised axial region: (0) absent, (1) present*.

43. *Anterior trunk segments greatly reduced forming a prothorax: (0) absent, (1) present*.

44. *Abdomen differentiated as limb-free segments: (0) absent, (1) present*.

45. *Abdomen differentiated as posteriorly-restricted segments: (0) absent, (1) present*.

46. *Posterior trunk segments with spinose posterior rim: (0) absent, (1) present*.

47. *Penultimate trunk segment elongate: (0) absent, (1) present*.

*Telson*

48. *Telson: (0) absent, (1) present*.

*Appendicular (pre-antennular, protocerebral)*

49. *Protocerebral appendages: (0) absent, (1) present*.

50. *Orientation of protocerebral appendages: (0) lateral, (1) ventral*.

51. *Sclerotization of protocerebral appendages: (0) absent, (1) present*.

52. *Fusion of protocerebral appendages: (0) absent, (1) present*.

53. *Hypostome with lateral slits: (0) absent, (1) present*.

54. *Protocerebral appendages spinose: (0) absent, (1) present*.

55. *Dorsal spine row: (0) absent, (1) present*.

56. *Ventral spine row: (0) absent, (1) present*.

57. *Relative orientation of dorsal spine row to podomere: (0) perpendicular, (1) lateral (chelate)*.

58. *Elongate ventral basal spine: (0) absent, (1) present*.

59. *Secondary (auxillary) spines on ventral spine row: (0) absent, (1) present*.

60. *Flexure of terminal protocerebral podomere: (0) ventral, (1) dorsal*.

*Appendicular (antennular, deutocerebral)*

61. *Deutocerebral appendage differentiated from posterior appendages: (0) absent, (1) present*.

*Appendicular (First-post-antennular, tritocerebral)*

62. *Tritocerebral appendage differentiated from posterior appendages: (0) absent, (1) present*.

63. *Basal podomere bearing endites: (0) absent, (1) present*.

64. *Appendages geniculate with distinct peduncle: (0) absent, (1) present*.

65. *Chelate (or subchelae) distal podomeres: (0) absent, (1) present*.

*Appendicular (post-tritocerebral)*

66. *Exites: (0) absent, (1) present*.

67. *Longitudinal wrinkling on exites: (0) absent, (1) present*.

68. *Exite fringed with setae: (0) absent, (1) present*.

69. *Exite and endopod derived from the same parent podomere (biramy): (0) absent, (1) present*.

70. *Trunk endopods: (0) absent, (1) present*.

71. *Endopod shape: (0) leg-like, (1) flap-like*.

72. *Sclerotization and arthropodization of trunk endopods: (0) absent, (1) present*.

73. *Endites on endopod: (0) absent, (1) present*.

74. *Endites on basal endopod segment: (0) absent, (1) present*.

75. *Endopod with terminal claws: (0) absent, (1) present*.

*Appendicular (lateral processes)*

76. *Posterior tagmata with elongate lateral processes: (0) absent, (1) present*.

77. *Lateral process pairs fused into a single element retaining evidence of original segmentation: (0) absent, (1) present*.

78. *Shape of lateral processes: (0) bulbous (unsclerotized) flaps, (1) cerci, (2) sub-triangular, (3) paddle-like*.

79. *Lateral telson processes recurved: (0) absent, (1) present*.

80. *Lateral telson processes spinose: (0) absent, (1) present*.

*Digestive*

81. *Orientation of mouth: (0) anterior, (1) ventral, (2) posterior*. [ADDITIVE]

82. *Circumoral structures: (0) absent, (1) present*.

83. *Nature of circumoral structures: (0) papillae, (1) sclerotized plates*.

84. *Lateral gut glands: (0) absent, (1) present*.
